# Exploring Strategies to Improve Adherence to Immunization Schedule: A Study among Children Attending Maternal and Child Health Clinic at Kenyatta National Hospital, Nairobi, Kenya

**DOI:** 10.1155/2020/4730205

**Published:** 2020-08-10

**Authors:** Esther Cheptanui Muathe, Mary Kamau, Eve Rajula

**Affiliations:** College of Health Sciences, School of Nursing Sciences, University of Nairobi, P.O. Box 19676-00202 Nairobi, Kenya

## Abstract

**Background:**

Globally, immunization is among the major contributors to public health, preventing 20% of childhood mortality annually. The highest fatality rates from vaccine preventable diseases are usually among children under five. Despite immunization guidelines put in place by the World Health Organization, globally, 1.5 million children die annually related to inadequate vaccination coverage. Existing literature indicate that there is an increase in nonadherence to immunization schedule in developing countries, and therefore, there is an increased demand to improve adherence to immunization schedule.

**Objective:**

To explore strategies that will improve adherence to immunization schedule among children under 24 months attending the Maternal and Child Health clinic at Kenyatta National Hospital.

**Methods:**

A cross-sectional mixed method study involving caregivers (*n* = 214) of well babies attending the Maternal and Child Health clinic. Data was collected using semistructured questionnaires, focus group discussions, and key informant interviews and analyzed using SPSS V.20.

**Results:**

There was a significant relationship between the level of education and marital status of the caregivers and adherence to immunization schedule. Barriers found that is related to adherence to immunization schedule included far distance from health facility, baby's sickness, and vaccine stock-outs while employment of a caregiver was a constrainer factor.

**Conclusion:**

The enabling factors to current strategies of improving adherence to immunization schedule were having more health facilities near residential areas, using text messages reminders a day before the clinic date to remind caregivers of the due date for the clinic, and constant availability of vaccines. The health system strategies that would improve adherence to immunization schedules were more flexible clinic hours, availability of vaccines on daily basis, phone call reminders by health care providers, and increasing awareness on the importance of both vaccinations and adherence to immunization schedule.

## 1. Introduction

Childhood vaccination is one among the many contributors of major global reductions in morbidity and mortality resulting from vaccine preventable diseases. It is an important intervention with an aim of reducing childhood morbidity and mortality as indicated in the Sustainable Development Goals [1]. It is estimated by the World Health Organization (WHO) and the United Nations Children's Emergency Fund (UNICEF) that 1.5 million children worldwide die from vaccine-preventable diseases annually [2]. This is caused by inadequate vaccination coverage and would otherwise be averted if immunization schedules are adhered to accordingly. Sub-Saharan Africa (SSA) and Southeast Asia contribute to the highest percentage of these deaths [2]. The WHO made great effort to reduce childhood morbidity and mortality by developing immunization guidelines which were adopted by Kenya by developing national guidelines on immunizations [3].

There are several childhood immunizable diseases and among them are tuberculosis, poliomyelitis, diphtheria, whooping cough, tetanus, pneumonia, measles, influenza, and rotavirus, among others. The Kenya Expanded Programme on Immunization (KEPI) was established in 1980 by the Ministry of Health (MOH) with an aim of immunizing all children in the country before their first birthday against the initial six under-five killer diseases which were tuberculosis, polio, diphtheria, whooping cough, tetanus, and measles. Over the years, more vaccines have been developed to provide immunity against hepatitis B, Haemophilus influenza type B, pneumonia, rotavirus, mumps, and rubella [3].

Immunization prevents an estimate of two to three million deaths resulting from life-threatening diseases among the under-fives annually [4]. Immunization has been made accessible to all the populations because it can be delivered through outreach activities [4]. Adherence to laid down vaccination schedules can significantly reduce the prevalence of vaccine-preventable diseases in children [5]. In a study done in Ethiopia in 2016, the main reason for nonadherence to immunization schedule was inadequate caregiver counseling leading to scanty information about vaccination schedules. Others were relationships between the health care providers and the clients which do not support information sharing, poor arrangement of immunization services, and lack of defaulter tracing systems [6].

The government of Kenya has made efforts to improve immunization coverage. However, adherence to immunization schedule has a gap. According to the immunization permanent register (MOH 510, 2017) in the Maternal and Child Health clinic, KNH, some caregivers take their children to the clinic weeks after the due date and others fail completely to take their children for immunization (Immunization Permanent Register MOH 510,2017). Therefore, the aim of this study was to explore strategies that will improve adherence to immunization schedule among children under 24 months attending the Maternal and Child Health clinic at Kenyatta National Teaching and Referral Hospital. The specific objectives were to assess enabling factors of the current strategies, assess barriers of current strategies, determine feasible strategies focusing on the caregiver, and determine achievable strategies focusing on the health system that will improve adherence to immunization schedule.

## 2. Materials and Methods

### 2.1. Study Setting

The study was conducted in the Maternal and Child Health Clinic (MCH) in Kenyatta National Hospital (KNH). The MCH is located at Clinic 66. It has a well-baby clinic where children less than 60 months are seen. Services offered are growth and development monitoring and childhood immunization as well as family planning services. The KNH is situated 4 km from the city center off Ngong road along Hospital Road. It occupies 45.7 hectares of land and within its complex is the College of Health Sciences of the University of Nairobi (UON) and other training and government institutions under the Ministry of Health. The Kenyatta National Hospital receives patients from various parts of the country as well as from East and Central Africa. Administratively, the hospital is divided into various departments according to different specialties. Maternal and child health (MCH) services is one among many services offered at KNH on an outpatient basis. The hospital is within Lang'ata Subcounty Public Health services of the Ministry of Health. As allocated by the Lang'ata District Public Health nurse, the coverage area for vaccination is the area within these boundaries. However, KNH being a national referral hospital receives self-referred clients from all over Nairobi and its neighboring counties like Kiambu, Machakos, and Kajiado. In addition, transit babies are also immunized, i.e., those who have been admitted to the paediatric wards and have not been immunized, as well as babies in the KNH maternity unit soon after they are born.

### 2.2. Study Design and Sampling

A descriptive cross-sectional mixed method study was conducted, which utilized both quantitative and qualitative approaches. The study involved sampled caregivers (*N* = 214) of children attending MCH, KNH. Sample size was calculated using Fisher's formula (2003). The calculation took into consideration the following: type 1 error which was fixed at 0.05, with a desire of a <5% chance of drawing a false positive conclusion, and a type 11 error that was set at a level of 0.20, meaning that the researcher desires a <20% chance of false-negative conclusion. Therefore, the power was set at 80%, representing the probability of avoiding a false-negative conclusion. The calculation established a sample size of 214 respondents who needed to have a consent to take part and assist in filling out the questionnaires. The immunization booklet was used to identify children who fell in the age category of 6 weeks to 24 months. Those who met the inclusion criteria were subsequently sampled until the desired sample size was achieved. For qualitative design, two focus group discussions (FGD) were conducted with a conveniently sampled group of caregivers of children attending MCH and two key informant interviews with the staff. The participants who participated in filling the questionnaires were exempted from the FGD. Each FGD is comprised of 10 participants. The key informants were two nurses who had worked in the clinic for more than five years. Respondents consented to the study after an explanation by the researcher and subsequently assisted in filling out the questionnaires. Participants for the FGD and key informants consented to participate in audio recording before taking part. The discussion was made possible by a predeveloped discussion guide.

### 2.3. Data Collection

Data were collected using a researcher-assisted structured questionnaire. The study instrument was pretested in the Maternal and Child Health clinic. Since the data collection procedure took only one month, there was no likelihood of including these participants in the study twice because the appointments are scheduled after every four weeks. The aim of this was to verify the data collection tool before data was collected and also to help estimate the time that would have been taken to administer the questionnaire to each respondent. The pretest results were used to improve the study tool for validity and reliability. The pretesting used 22 respondents which was 10% of the study sample size. Qualitative data was collected using an interview guide to guide FGD. The guide had open questions which enabled the participants to express themselves exhaustively. A key informant interview guide was used to interview the two nurses who had worked in the clinic for more than five years. The guide had two sections. Section A covered social demographic data and section B had open-ended questions about the clients seen in the clinic. The parents' questionnaire was in English and Swahili versions, and the interview was conducted in English or Kiswahili where applicable.

### 2.4. Data Analysis

Questionnaires were verified for completeness at the end of each day of data collection. A unique code for each questionnaire was entered into a Microsoft Excel spreadsheet where data cleaning was done. Identification of missing/extreme values and inconsistencies were done and corrected. Incomplete and wrongly answered questionnaires were omitted during data entry process. Data were analyzed using a computer software (SPSS V 20) whereby descriptive and inferential outputs were generated. Descriptive data was reported using mean, frequencies, and proportions. Associations between variables were done using Pearson's Chi-square. Qualitative data were transcribed and translated. Analysis was done manually by reviewing the field notes and listening to the audiotapes and grouping the research findings as per the study objectives. Different positions emerging under study objectives were noted and a summary written.

### 2.5. Ethical Consideration

The research proposal was reviewed for approval by the Kenyatta National Hospital/University of Nairobi Ethics and Research committee (approval number P66/02/2019). Permission to collect data was granted by the pediatric department administration (Ref. KNH/PAEDS.HOD/48 Vol.II). Participants were informed about the study purpose following which verbal and written consent was obtained from them. The final results have been shared with MCH in charge and hospital decision makers for action to be taken appropriately.

## 3. Results

### 3.1. Caregiver Social Demographic Characteristics

Majority of the participants in the study were mainly parents of the enrolled children. Majority, 92.2%, of those who escorted their children to the clinic were mothers, 6.1% (*n* = 13) were fathers while the smallest percentage 1.9% were brought by other relatives. The age category of caregivers ranged from 19 to 49 years with the mean age being 30 years. Age distribution showed that most (47.9%) of the caregivers were aged 26 to 30 years. Regarding the marital status, majority, 86.8%, of the caregivers were married, and the rest were either single (10.4%), widowed (2.4%), or separated/divorced (0.5%). The highest percentage of the caregivers 55.1% lived in Nairobi County, while the rest 44.9% lived in the neighboring counties. With respect to education level, those having university/college level of education had the highest percentage 56.1%, while 37.7% had attained secondary school level of education and 6.1% primary level. Regarding occupation, those with salaried employment, 47.1%, were the highest. The rest were either self-employed (43.6%), casual labourers (7.1%), housewife/not employed (10%), or student (0.5%). Concerning household gross monthly income, 24.6% of the caregivers were earning a gross monthly income of above 400 USD (KSh.40, 000), 20.9% earned between 210 and 300 USD (KSh.21, 000 and 30, 000), 21.8% earned between 110 and 200 USD (KSh.11, 000-20,000), while those who earned less than 100 USD (KSh.10, 000) were 22.7%. Time taken by most of the caregivers to reach the nearest immunization clinic is 2-3 hours (66.5%), while the rest took less than 1 hour (33.0%) and over 5 hours (0.5%), respectively. Most of the participants 42.2% used between 1 and 2 USD (KSh. 100-200) as bus fare to reach home, while the rest 28.4% spent less than 1 USD (<KSh.100), 21.3% spent above 2 USD (>KSh. 200) and 8.1% used private means (Table 1).

### 3.2. Relationship between Caregiver Social Demographics and Adherence to Immunization Status

The status of adherence to immunization schedule was determined in this study. When verified with the immunization booklet/card, the highest percentage (54.9%) of the children had been immunized as scheduled while closing being followed by those who had not had not received immunizations as per the schedule (45.1%). The factors associated with adherence to immunization schedule were the level of education and marital status of the caregiver. There was a significant relationship between the level of education of the caregivers and adherence to immunization schedule (OR = 0.886, 95%CI = 0.566-1.386; *P* = 0.002).Those who had college/university level of education (60.5%) were more likely to adhere to immunization schedule than caregivers with primary level (15.4%) and secondary level of education (41.3%). Similarly, marital status was statistically significantly associated with adherence to immunization schedule (OR = 2.299, 95%CI = 1.022-5.172; *P* = 0.046). Caregivers who are single had increased nonadherence to immunization schedule (63.6%) as compared to those who are married (44.6%) (Table 2).

### 3.3. Enabling Factors of the Existing Strategies of Improving Adherence to Immunization Schedule

The caregivers gave recommendations that would act as enabling factors to the already available strategies of improving immunization schedule and the greatest number, 48.1%, recommended that there should be more health facilities near residential areas. About a quarter, 27.4%, said that vaccines should be available always and similarly, 23.6%, said they should be reminded on return date using text messages. The other recommendations (5.8%) were flexible clinic hours and making a follow-up when they fail to come to the clinic. In the focused group discussion, similar recommendations were given. The caregivers gave the following enabling factors: The respondents suggested some mechanisms for facilitating timely immunization. These include having services brought closer to their places of residence, reminders from nurses such as text messages or phone calls, putting alarms/reminders on their phones, and referring to the immunization booklet more frequently. Related excerpts from the respondents include the following:.

“Government should consider building a government hospital near our place” (FG 1, Participant 2).

“Text messages or phone calls from nurses will help us in remembering the clinic date” (FGD 2, Participant 7).

### 3.4. Barriers of Current Strategies of Improving Adherence to Immunization Schedule

Thirty-seven percent of the participants pointed out lack of time off to take children for immunization due their busy form of employment causing their failure to adhere to the immunization schedule. Employment is thus a constrainer factor. A barrier of access was reported by 29.2% who said their home was far from the health facility, while 25.5% reported baby's sickness and 3.8% reported vaccine stock-out as a barrier. The other barriers (8.5%) to immunization schedule which came up in the study were caregiver getting late to the clinic, too demanding jobs, forgetfulness, lack of bus fare, and traffic jam. These findings are also supported by the focus group discussions (FGD) as stated below:.

“Sometimes I lack bus fare. Where I live there is no government hospital nearby and the nearest is either Kiambu or Kenyatta. If the clinic date falls on the time of the month when I have no money, I have to wait till I get so that I bring the baby to the clinic.” (FGD 1, Participant 6).

“I get preoccupied and I forget only to remember when the due date has passed. The work I.

do occupies me the whole day and sometimes I work even on weekends”. (FGD 1, Participant 3).

“Baby's illness. When the baby is on treatment, the baby has to complete treatment first.

before continuing with vaccinations” (FGD 2, Participant 8).

Other quotes were: “lack bus fare”, “no government hospital nearby”, “no money”, “I forget”, “find when vaccines are over”.

The above quotes were also supported by one key informant who mentioned that the key barrier was a long distance to the facility where vaccination was available and therefore clients arrive when clinic is already closed.

### 3.5. Feasible Strategies Focusing on the Caregiver That Will Improve Adherence to Immunization Schedule

Each caregiver had a way of remembering the scheduled date. Most of them, 60.5%, stated that they refer to the immunization card and 20.5% use the phone to remind them. Another 11.9% use the diary as a reminder while 7.6% used other strategies such as marking the date on the calendar, asking a family member to remind them, and choosing a specific date on each month. The other strategies which came up in the study were informing the employer two days before that the clinic date is almost due and asking a relative or neighbor to take care of other children as the caretaker escorts the baby to the clinic.

This was also supported by the following quotes from the focus group discussion (FGD):.

“By having a small baby, it is important for each mother to keep updating her reminder on the phone”. (FGD 2, Participant 1).

“I can use my phone reminder, so the alarm goes on three days before the clinic date”. (FGD 1, Participant 10).

### 3.6. Achievable Strategies Focusing on the Health System That Will Improve Adherence to Immunization Schedule

The achievable strategies recommended by study respondents that were focused on the health system were as follows: Fifty-seven percent of the participants suggested that phone call reminders would help them remember the clinic date, 62.6% gave a suggestion that the clinic should run for more hours per day, 8.5% said that vaccines should always be available in the clinic, while 0.5% gave a suggestion that health care providers should pay them a visit in their homes.

### 3.7. Determinants of Adherence to Immunization Schedule

To identify factors associated with adherence to immunization schedule among caregivers, a multiple regression analysis was performed. Only two factors met the criteria to be considered influencing adherence (Table 2). Respondents with tertiary level of education were 1.9 times more likely (OR = 1.9; 95%CI = 0.566-1.386; *P* = 0.002) to adhere to the immunization schedule compared to those with lower education level. Respondents who were married were 2.3 times more likely (OR = 2.299; 95%CI = 1.022-5.172; *P* = 0.046) to adhere to the immunization schedule as compared to those who were single (Table 3).

## 4. Discussion

This study sought to explore strategies that will improve adherence to the immunization schedule among children under 24 months attending the Maternal and Child Health clinic. When verified from the immunization booklet/card, most (54.9%) of the children had been immunized as scheduled while another large group (45.1%) had not received immunizations as per the schedule. From these findings, it can be concluded that a large group of children do not adhere to the immunization schedule. The factors which determined the adherence to immunization schedule were the level of education and marital status of the caregiver. The main reasons attributed to nonadherence as mentioned by participants in FGD included lack of bus fare, employment of the caregiver being very demanding, baby's sickness, getting late to the clinic and missing immunization services, and caregiver forgetting the return date.

The young adult age category of 26-30 years dominated the study. Majority of the caregivers learned beyond primary school. Since majority of the caregivers were mothers, this shows the acknowledgement of the girl-child education by majority of Kenyans. This should be enhanced even more to help improve adherence to immunization schedule, as demonstrated in this study that those with higher levels of education were more likely to adhere to immunization schedules compared to those with lower education level. Moreover, this age category is comprised of the caregivers, majority of whom had some form of employment. However, they stated that they missed the immunization schedule because of their involving activities at their workplace. Thus, this study shows that employment is a constrainer factor in relation to nonadherence to the immunization schedule. Strategies to improve adherence to immunization schedule such as flexible clinic hours or increasing clinic hours are vital in addressing this gap.

### 4.1. Association between the Caregiver's Sociodemographics and Nonadherence to Immunization Schedule

The caregiver's level of education significantly influenced adherence to the immunization schedule. There was an increased nonadherence to immunization schedule among caregivers with primary level of education and secondary level as compared to those who had college/university level of education. The study findings support previous findings in a study done in India which found that low adherence to immunization schedule has been associated with social demographic characteristics of the parents/caregivers which range from family size to the level of caregiver education [7]. One study also cited factors for incomplete vaccination as maternal education, as well as socioeconomic status of the family [8]. This implied that low level of education could have led to the caregivers' lack of awareness of the negative outcomes of nonadherence to immunization schedule. It could also be due to the fact that caregivers who are educated have more knowledge about good medical practices. They could have, during their training, come across courses on healthcare. The study showed that 22.7% of the respondents had a gross monthly income of less than KSh.10, 000. This implies that they had inadequate economic empowerment, and this could lead to dependence on others economically. Consequently, this contributes to missing immunization schedules due to lack of bus fare as stated by majority of caregivers. This incident can easily be prevented if more health facilities were available near residential areas.

The marital status of the caregiver also significantly influenced adherence to the immunization schedule. Our study showed that single parents had a greater nonadherence status as compared to those who were married. In a study done in Ghana, it was found out that the people that are more likely to influence attendance to immunization clinic include a healthcare worker and a supportive spouse [18]. Single parenthood could have contributed to nonadherence to the immunization schedule due to lack of support. As regards the place of residence, a big number of the caregivers live outside Nairobi County and this is the reason why most of them stated that they missed immunization scheduled date because they arrived to the clinic late. In this regard, building more government health facilities near residential areas is important as this will reduce the time taken to reach the immunization clinic.

### 4.2. Enabling Factors of the Current Strategies of Improving Adherence to Immunization Schedule

Twenty-four percent (*n* = 50) of the caregivers recommended that text messages should be used to remind them of the return date for immunizations. Previous studies supported this strategy. Mobile phone-based reminder has been considered a useful strategy in rural Western Kenya to have clients remember their due immunization dates [9]. A study done in USA showed that technology-based interventions in health care has demonstrated great potential to transform vaccine delivery support and improve immunization coverage in the United States [10].

Reduction of missed opportunities by health care providers can be enhanced by provider-based interventions which include client reminders/recall, assessment of the knowledge level of each mother, and education of clients [11], as evidenced in this study too.

### 4.3. Barriers to Current Strategies of Improving Adherence to Immunization Schedule

Thirty-seven (*n* = 78) of the respondents cited employment as a barrier to adherence to the immunization schedule. One study also cited factors for incomplete vaccination as fear of losing daily employment [8]. Another study by [12] pointed out a barrier to adherence to the immunization schedule as primary care providers being busy with other obligations and only arrive to the clinic when it is late. One key informant who mentioned during the interview that the key barrier to the immunization schedule was the long distance to the health facility and therefore clients arrive when clinic is already closed supported this.

Twenty-nine percent (*n* = 62) of the caregivers said their home was far from the health facility. This is supported by other studies which stated that one known barrier to utilization of healthcare is the long distance to healthcare facilities [13] and that health system factor which include long distance to the health facility [7] also plays a role in nonadherence to the immunization schedule.

One key informant who mentioned that the key barrier was a long distance to the facility where vaccination was available and therefore clients arrive when clinic is already closed also supported this. With these results, these barriers can be eradicated by having more health facilities near residential areas and also flexible immunization hours, as indicated by the respondents in this study.

### 4.4. Feasible Strategies Focusing on the Caregiver That Will Improve Adherence to Immunization Schedule

With regard to strategies focusing on the caregiver, the study revealed that most (60.5%) of the respondents stated that they refer to the immunization card as a way of remembering the clinic date. This is not a reliable strategy because they can easily forget. Caregivers should be encouraged to get a more reliable strategy like phone alarm reminder which is used by a few (20.5%). This was supported by a study done in Nigeria by [14] which showed that the reminder system can improve client's adherence to health services including immunization. In a study done in Kenya, forgetfulness of the caregiver due to preoccupation with other activities was found to contribute to nonadherence to the immunization schedules [15]. Reminders help to inform parents/caregivers of the due date for their children's vaccination [11]. In this study, some caregivers gave forgetfulness as a reason for missed schedules. In a previous study, it was observed that two main causes of missed vaccinations were that the prior reminders were not sufficient to parents and parents' forgetfulness. Another study found out that reminders to vaccination are effective in increasing immunization rates [5]. Hence, there is a need to employ this strategy routinely to improve adherence to immunization schedules.

### 4.5. Achievable Strategies Focusing on the Health System That Will Improve Adherence to Immunization Schedule

A total of 132 (62.6%) caregivers gave a suggestion that immunization clinics should run for more hours per day. This would be a good strategy of improving adherence to the immunization schedule especially if there were flexible immunization hours. This is supported by the statement by [16] in a study done in India. The study stated that as the countries are embarking upon journey towards Universal Health Coverage (UHC), the learning and initiatives for scaling up coverage in immunization programs combined with health system approach should be optimally utilized for expansion of other health interventions.

Caregivers mentioned long distance to health facility as a hindrance to adherence to the immunization schedule. This presents a barrier of access to immunization services. A previous study done in Kenya focused on demand side factors including distance to the health facility as a hindrance to completion of immunizations (14). A good number, 11.8%, of caregivers pointed out in the study that they did not receive adequate information concerning adherence to immunization schedule. This is supported by a study done in Bangladesh. In this study, dropout rates were heightened by inadequate information about the immunization schedule [17]. In an effort to improve adherence to the immunization schedule, one of the strategies focusing on health systems is providing adequate information to caregivers either as groups or one on one, as indicated by this study.

## 5. Conclusion

This study concludes that the enabling factors to current strategies of improving adherence to the immunization schedule were having more health facilities near residential areas to avoid travelling long distances to seek immunization services, using text message reminders a day before the clinic date to remind caregivers on the due date for the clinic, and constant availability of vaccines. The study found barriers to current strategies of improving adherence to the immunization schedule to be the far distance residence from health facility, baby's sickness, and vaccine stock-outs. Employment was found to be a key constrainer factor to adherence to the immunization schedule. Findings on the feasible strategies focusing on the caregiver as reported by most caregivers was a better way of remembering the clinic date like phone alarm reminder instead of referring to the immunization booklets, since most caregivers mentioned forgetfulness due to preoccupation with other activities as a hindrance to adherence to the immunization schedule. Finally, the study found that the achievable strategies focusing on the health system that will improve adherence to the immunization schedule were more flexible clinic hours and running immunization clinics for more hours in a day, availability of vaccines on daily basis, phone call reminders by health care providers, and creating awareness on the importance of vaccinations and more so on the importance of adhering to the immunization schedule. These provide possible solutions for health facilities and decision makers to improve immunization adherence.

## Figures and Tables

**Table 1:** 

Social demographic characteristics	Frequency (*n* = 214	Percentage (%)
Age in years		
Mean age (+/-SD)	30+/-1	
25 years and below	32	15.4
26-30	102	47.9
Above 30 years	79	37.1
Relationship with the child		
Mother	195	92.0
Father	13	6.1
Guardian	4	1.9
Marital status		
Single	22	10.4
Married	184	86.8
Widowed	5	2.4
Separated/divorced	1	0.5
Residence (county)		
Nairobi	118	55.1
Neighboring	96	44.9
Highest level of education		
Primary level	13	6.1
Secondary level	80	37.7
College/university level	119	56.1
Occupation		
Student	1	0.5
Housewife/not working	2	0.9
Casual laborer	15	7.1
Self-employed	92	43.6
Salaried employment	101	47.9
Gross family income (KSh.)		
<10, 000	48	22.7
11,000-20,000	46	21.8
21,000-30,000	44	20.9
31,000-40,000	24	11.2
>40,000	52	24.6
Time taken to reach health facility		
<1 hour	70	33.0
2-3 hours	141	66.5
>5 hours	1	0.5
Amount of bus fare used		
<100	60	28.4
100-200	89	42.2
200	45	21.3
Private means	17	8.1

**Table 2:** Relationship between social demographic characteristics of caregivers and adherence to immunization schedule.

Sociodemographic characteristics of caregivers		Did child get immunizations as scheduled? Refer to immunization card/booklet		
		Yes	No	
		%	%	*P* value
Relationship with the child	Mother	55.7	44.3	0.784
	Father	46.2	53.8	
	Guardian	50.0	50.0	
	Other	0.0	0.0	

Age	25 years and below	67.7	32.3	0.276
	26-30 years	55.4	44.6	
	Above 30 years	49.4	50.6	

Marital status	Single	36.4	63.6	0.046
	Married	55.4	44.6	
	Widowed	0.0	100.0	
	Separated/divorced	0.0	100.0	

Highest level of education	Primary level	15.4	84.6	0.002
	Secondary level	41.3	58.8	
	College/university level	60.5	39.5	
	Have no formal education	0.0	0.0	

Occupation	Student	0.0	100.0	0.074
	Housewife/not working	100.0	.0	
	Casual laborer	26.7	73.3	
	Self-employed	54.3	45.7	
	Salaried employment	59.4	40.6	

Gross income per month	<10,000	45.8	54.2	0.672
	11,000-20,000	58.7	41.3	
	21,000-30,000	56.8	43.2	
	31,000-40,000	61.9	38.1	
	>40,000	53.8	46.2	

Time taken to reach the nearest immunization clinic and back home	<1 hour	55.7	44.3	0.539
	2-3 hours	54.6	45.4	
	4-5 hours	.0	.0	
	>5 hours	.0	100.0	

Money used as bus fare to reach the clinic and back home	<100/-	51.7	48.3	0.522
	100-200/-	52.8	47.2	
	Above 200/-	57.8	42.2	
	Private means	70.6	29.4	

**Table 3:** Determinants of adherence to immunization schedule.

	Coefficient	S.E. of coefficient	p-value	OR	95% CI for OR	
					Lower	Upper
Marital status Single Married Separated/divorced	0.832	0.414	0.044	2.299	1.022	5.172
Highest level of education Primary Secondary Tertiary	-0.121	0.229	0.595	0.886	0.566	1.386

## Data Availability

The data used to support the findings of this study are available from the corresponding author upon request.
